# Association of TNF-α genetic variants with neonatal bronchopulmonary dysplasia: consolidated results

**DOI:** 10.3389/fped.2024.1511355

**Published:** 2024-12-19

**Authors:** Seyedeh Elham Shams, Seyed Alireza Dastgheib, Seyede Arefe Mousavi-Beni, Mohamad Hosein Lookzadeh, Seyed Reza Mirjalili, Mohammad Golshan-Tafti, Reza Bahrami, Maryam Yeganegi, Amirhossein Shahbazi, Ali Masoudi, Amirmasoud Shiri, Mahmood Noorishadkam, Hossein Neamatzadeh

**Affiliations:** ^1^ Department of Pediatrics, Hamadan University of Medical Sciences, Hamadan, Iran; ^2^ Department of Medical Genetics, School of Medicine, Shiraz University of Medical Sciences, Shiraz, Iran; ^3^ Afshar Hospital Cardiovascular Research Center, Non-Communicable Disease Research Center, Shahid Sadoughi University of Medical Sciences, Yazd, Iran; ^4^ Mother and Newborn Health Research Center, Shahid Sadoughi University of Medical Sciences, Yazd, Iran; ^5^ Department of Pediatrics, Islamic Azad University of Yazd, Yazd, Iran; ^6^ Neonatal Research Center, Shiraz University of Medical Sciences, Shiraz, Iran; ^7^ Department of Obstetrics and Gynecology, Iranshahr University of Medical Sciences, Iranshahr, Iran; ^8^ Student Research Committee, School of Medicine, Ilam University of Medical Sciences, Ilam, Iran; ^9^ Student Research Committee, School of Medicine, Shahid Sadoughi University of Medical Sciences, Yazd, Iran; ^10^ Student Research Committee, School of Medicine, Shiraz University of Medical Sciences, Shiraz, Iran

**Keywords:** premature lung diseases, bronchopulmonary dysplasia, TNF-α, preterm, neonate

## Abstract

**Objectives:**

Inflammation is increasingly recognized as a key factor in the pathophysiology of bronchopulmonary dysplasia (BPD). While previous research has established significant links between TNF-α polymorphisms and BPD susceptibility, further validation of these associations is needed. This study aims to examine the relationship between TNF-α polymorphisms and the risk of BPD.

**Methods:**

All relevant articles published before October 1, 2024, have been screened in the PubMed, Web of Science, CNKI, and Scopus databases.

**Results:**

A total of 14 case-control studies were conducted: five studies with 1,252 cases and 1,377 controls on −308G/A, three studies with 1,180 cases and 1,194 controls on −238G/A, four studies with 149 cases and 192 controls on −857C/T, and two studies with 82 cases and 162 controls on 1,031 T/C. A significant association was found between the TNF-α −238G/A polymorphism and the risk of BPD. However, no significant relationships were observed for the TNF-α −308G/A, −857C/T, and 1,031 T/C polymorphisms regarding BPD susceptibility.

**Conclusions:**

Our findings indicate a significant association between the TNF-α −238G/A polymorphism and the susceptibility to BPD in preterm neonates, suggesting potential biomarkers for its pathogenesis. However, this meta-analysis has limitations, including possible publication bias and heterogeneity due to the limited number of studies, which may affect the reliability of our conclusions. Moreover, population variability further complicates the interpretation of the link between TNF-α polymorphisms and BPD risk.

## Introduction

Bronchopulmonary dysplasia (BPD), also known as chronic lung disease, is one of the serious complications in extremely preterm (<28 weeks of gestational age) and very low birth weight (<1,500 g) neonates, which has a negative impact on the prognosis of the infants (1, 2). BPD results from an imbalance between lung injury and repair caused by complex interactions between various environmental factors and genetic susceptibility in the developing lung. It is clinically characterized by the need for supplemental oxygen therapy and/or mechanical ventilation for greater than 4 weeks (mild) or over 36 weeks postmenstrual age (moderate and severe) (3, 4). Nonetheless identification of BPD over half a century as well as advanced perinatal and neonatal care, there has been no amelioration in the occurrence, morbidity, mortality, and severity of BPD (5, 6). Moreover, the economic burden of BPD on family and health systems remains largely unknown and is regarded as one of the exorbitant morbidities of preterm birth (7, 8). An appraisal estimation of charges including care during neonatal intensive and related morbidities, reported a median total cost of $373,468 for neonates with BPD (9). Therefore, prevention strategies could help to minimize costs and conserve healthcare resources (9, 10). The reported incidence of BPD in the literature varies considerably, in part owing to differences in racial or ethnic background of the preterm neonates, referral patterns or treatment practices, but also to differences in BPD definitions (11–13). The survey results showed that among premature infants aged 22–29 weeks, the incidence of mild to moderate BPD was 36.9%, and the occurrence of severe BPD was 3.7% (14–16). Even after half a century of investigations, the pathogenesis of BPD is not fully realized (17). The pathogenesis of BPD involves a variety of factors and its incidence and severity increase as gestational age and birth weight decreases. The evaluated antenatal risk factors of severe BPD included gender, iatrogenic preterm birth, gestational hypertension, low GA, and small for gestational age (below 10th percentile for gestational age) birth weight (13, 14, 17–19).

Twins studies have indicated high substantial concordance rates for BPD: 3.69% for MZ and 1.4% for DZ twins (20, 21). Bhandari et al., estimated that hereditary factors explained 53% of the discrepancy in BPD in premature neonates by sub-analyses (21). In recent years, epigenome-wide association studies (EWAS) and genome-wide association studies (GWAS) have determined several loci with a different effect on BPD development in extremely low birth weight infants; such as surfactant proteins (SP-A, SP-B, SP- C) genes, Hedgehog signaling pathway genes, Toll-like receptors (TLR10, TLR1, TLR4), Oxidative stress-related genes, matrix metalloproteinase (MMPs), nitric oxide synthase (NOS2), C-reactive protein (CRP), lipopolysaccharide-binding protein (LBP), Cathepsin H and Osteonectin (22–25). Nevertheless, there have been no certain genes identified for BPD pathogenicity (26). Studies have shown that inflammatory mechanisms, cytokine functions, non-coding RNA and signal pathway factors are involved in the pathogenesis of BPD in preterm neonates (27, 28). Mechanical ventilation can activate the epidermal growth factor receptor in lung tissue and induce an increase in inflammatory cytokines in animals (29). The interaction between genetic liability loci and environmental factors is an essential mechanism for development of BPD (30). Environmental risk factors, such as hyperoxia and inflammation resulting to abnormal gene expression have assisted to the development of BPD (31, 32).

To date, no specific gene has been definitively identified as the causative factor for BPD in neonates, and current research findings have been challenging to reproduce. However, genes involved in inflammation-related pathways, such as tumor necrosis factor-α (TNF-α) and various interleukins, have been extensively studied in relation to several lung diseases, including acute lung injury, bronchial asthma, pulmonary fibrosis, and chronic obstructive pulmonary disease (33–35). TNF-α is known to have a protective role during early fetal development but tends to trigger negative inflammatory responses after birth. It regulates proteins related to TNF receptors, such as TNFR-correlated factor 1 (TRAF1) (36), in lung cells. This cytokine is crucial in the inflammatory processes associated with BPD in preterm infants, as elevated levels of TNF-α have been found in the tracheal aspirates and serum of these infants shortly after birth, highlighting its involvement in the chronic inflammation linked to BPD (37). Genetic variations, like the TNF-α−238 polymorphism, are associated with differences in TNF-α production; infants carrying the adenine allele display lower TNF-α levels and a reduced risk of severe BPD (38). In contrast, the TNF-α−308 polymorphism has shown inconsistent relationships with BPD risk (39). These genetic factors underscore the complexity of the inflammatory response, where increased TNF-α can worsen lung injury, while lower levels may provide protective effects. The influence of ethnic differences is notable, with significant associations reported in African American populations (40) but inconsistent findings in other groups, including Caucasian (41, 42) and Korean (43) preterm infants. Overall, although current evidence suggests that TNF-α plays a crucial role in BPD development and severity, inconsistencies in study results underline the need for comprehensive, multicenter meta-analyses to elucidate the relationship between TNF-α genetic polymorphisms and BPD susceptibility, enabling personalized treatment strategies for high-risk preterm infants.

## Materials and methods

### Search strategy

An extensive search was conducted across several online databases for case-control studies examining the correlation between TNF-α polymorphisms and BPD published up until October 1, 2024. The databases included PubMed, ResearchGate, Web of Science, Europe PMC, Elsevier, Cochrane Library, EMBASE, SciELO, Google Scholar, Wanfang Data Company, Chaoxing, Chinese Medical Citation Index (CMCI), VIP Information Consulting Company (VIP), Chinese Medical Current Contents (CMCC), Chinese Biomedical Database (CBD), Sinomed, MEDREX, China/Asia On Demand (CAOD), Asia Document Delivery, Baidu, and Chinese National Knowledge Infrastructure (CNKI) Weipu Periodical Database. Additionally, databases such as PsycINFO, Scopus, and ClinicalTrials.gov were also considered. The search strategy employed a combination of keywords including (“Chronic Lung Disease” OR “Bronchopulmonary Dysplasia” OR “Lung Disease” OR “Premature Lung Injury”) AND (“Infant” OR “Neonates” OR “Preterm” OR “Newborn Infant” OR “Infant Preterm” OR “Preterm Infants” OR “Neonatal Prematurity” OR “Low Birth Weight” OR “Infants with Low Birth Weight” OR “Underweight Infants”) AND (“Tumor Necrosis Factor alpha” OR “TNF-α” OR “Cytokine” OR “Inflammatory Mediators”) AND (“Gene” OR “Genetic” OR “DNA” OR “Single-Nucleotide Polymorphism” OR “SNPs” OR “Polymorphism” OR “Genotype” OR “Allele Frequency” OR “Mutation” OR “Variant” OR “Genetic Variation”). Furthermore, manual searches of bibliographies from the retrieved studies and relevant reviews were conducted to identify additional suitable papers. Selected articles were published in English, Russian, and Chinese. Since this meta-analysis did not involve participants, obtaining informed consent was not required.

### Including and excluding criteria

All studies in this review met specific eligibility criteria: (1) Only case-control or cohort studies published in English, Russian, or Chinese were included; (2) Studies had to examine the association between TNF-α polymorphisms and the risk of BPD; (3) The case group comprised premature infants diagnosed with BPD, while the control group included non-BPD premature infants; (4) Studies needed to provide sufficient data to calculate odds ratios (ORs) and 95% confidence intervals (CIs). Exclusion criteria were: (1) Case series, commentaries, editorials, letters, reviews, animal studies, *in vitro* experiments, conference abstracts, and meta-analyses were excluded; (2) Research with incomplete data or studies from which the original manuscript could not be retrieved, even after contacting the authors, were excluded; (3) Studies lacking adequate or accessible data for analysis were not included; (4) Research focusing on family members, such as linkage studies, sibling analysis, or studies involving monozygotic and dizygotic twins, were excluded; (5) Studies with overlapping data or duplication were not considered.

### Data extraction

Two investigators independently reviewed the bibliographies, gathered data, and cross-checked their findings based on the inclusion and exclusion criteria. Any disagreements were resolved through discussion or by involving a third scientist. During the literature screening, titles and abstracts were first reviewed to eliminate irrelevant studies, followed by a full-text read to confirm inclusion. The following key information was extracted from studies that met the criteria: first author name, publication date, country of origin, ethnic background, genotyping methods, total cases and controls, genotype frequencies for available TNF-α polymorphisms, Hardy-Weinberg equilibrium (HWE) test results, and minor allele frequencies (MAFs) in non-BPD infants. If multiple studies were authored by the same investigator(s) with duplicate records, only the most recent publication or the one with the largest sample size was included.

### Statistical analysis

To evaluate the correlation between TNF-α polymorphisms and the risk of BPD, ORs with corresponding 95% CIs were calculated. Statistical significance for the combined data was determined using the Z-test to assess the disparity between the population mean and the sample mean. This meta-analysis encompassed five genetic models: allelic (B vs. A), homozygous (BB vs. AA), heterozygous (BA vs. AA), dominant (BB + BA vs. AA), and recessive (BB vs. BA + AA). The chi-square test, recognized as the predominant method for evaluating heterogeneity significance, was applied with a significance level of *p* < 0.05. According to Cochrane guidelines, heterogeneity among studies was quantified using the I^2^ statistic, with thresholds ranging from 0% to 100%. A random-effects model (DerSimonian-Laird method) was employed if I^2^ exceeded 50%, otherwise, a fixed-effect model (Mantel-Haenszel method) was utilized for analysis (44). Sensitivity analyses were conducted by sequentially omitting one study at a time to evaluate the robustness of the results. Publication bias was assessed using Begg's test, which involved plotting the standard error (SE) of each study against its OR, along with Egger's test and visual inspection of funnel plot asymmetry. Should publication bias be identified, the trim-and-fill method was applied to adjust the conclusions accordingly (45). The HWE was assessed using the Chi-Square (*χ*^2^) statistic in healthy subjects from a single study, with a significance threshold set at a *p*-value of less than 0.05. Data synthesis from the primary studies was performed using the Comprehensive Meta-Analysis software (Version 4.0, Biostat, USA). A two-sided *p*-value of less than 0.05 was deemed statistically significant.

### Newcastle-Ottawa scale

The Newcastle-Ottawa Scale (NOS) is a widely respected tool for evaluating the quality of non-randomized studies, especially in meta-analyses. It assesses studies based on three criteria: participant selection, group comparability, and outcome assessment, which includes exposure ascertainment and outcome measurement. Each study can receive up to nine stars, with higher scores reflecting better methodological quality. The NOS systematically evaluates bias risk, boosting the credibility of meta-analysis findings. By using the NOS, we rigorously assessed the studies included in this meta-analysis, leading to a more reliable synthesis of evidence for our research question.

### Selected study characteristics

**Figure 1:**
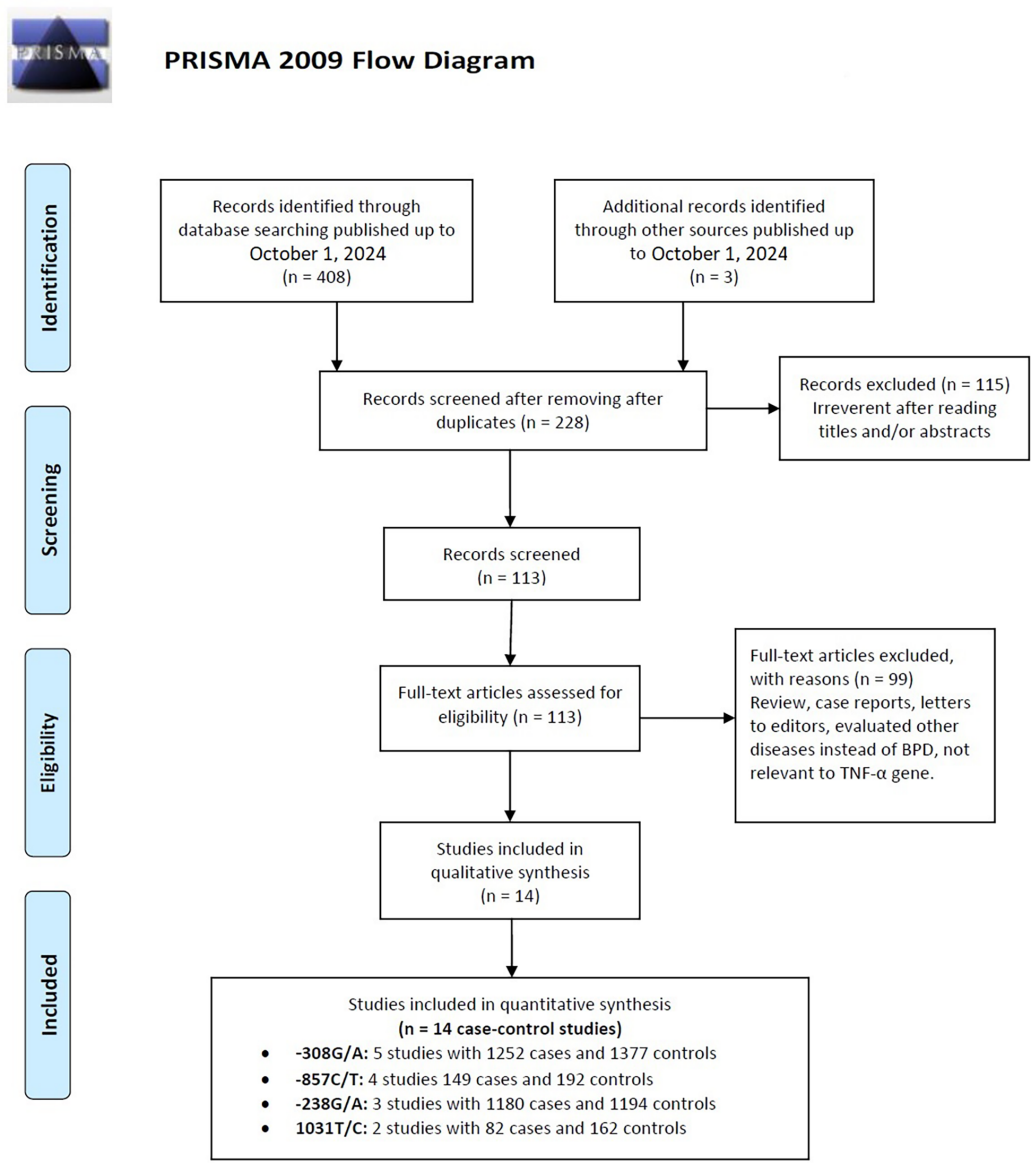
Flow diagram of the study selection process, detailing the number of studies identified, screened, assessed for eligibility, and included in the final analysis.

Flowcharts illustrating the detailed selection process are presented in Figure 1. Initially, a total of 411 papers were identified through various electronic databases, followed by a thorough manual screening of the full contents. After reviewing the titles and abstracts, 183 duplicate records and 115 irrelevant studies were excluded, resulting in 113 articles for which full texts were read. Ultimately, 14 case-control studies from eight independent publications (41–43, 46–50) were included in this meta-analysis, comprising a total of 2,663 neonates diagnosed with BPD and 2,925 neonates without BPD. The characteristics of the included studies are summarized in Table 1. Specifically, five studies involved 1,252 cases and 1,377 controls concerning the −308G/A polymorphism; three studies included 1,180 cases and 1,194 controls for the −238G/A variant; four studies analyzed 149 cases and 192 controls regarding the −857C/T polymorphism; and two studies focused on 82 cases and 162 controls for the 1,031 T/C variant. These studies were published between 2005 and 2017, written in English or Chinese. Genotyping of the polymorphisms was performed using three methods: polymerase chain reaction (PCR), TaqMan, and PCR-restriction fragment length polymorphism (PCR-RFLP). Research was conducted across multiple countries, including Taiwan, Germany, Korea, Poland, China, and Egypt. Among the included studies, nine were conducted with Asian neonates, four with Caucasian neonates, and one with African neonates.

**Table 1:** Study characteristics in the meta-analysis.

First author/year	Ethnicity (country)	Genotyping methods	Case/control	Cases					Controls					MAFs	HWE	NOS
				Genotypes			Alleles		Genotypes			Alleles				
−308G/A				GG	GA	AA	G	A	GG	GA	AA	G	A			
Lin 2005	Taiwan(Asian)	NA	112/112	82	24	6	188	36	70	36	6	176	48	0.214	0.630	7
Mailaparambil (42)	Germany(Caucasian)	PCR-RFLP	44/107	33	10	1	76	12	75	28	4	178	36	0.168	0.501	6
Seung Jo (43)	Korea(Asian)	TaqMan	38/55	30	8	0	68	8	46	9	0	101	9	0.082	0.508	6
Szpecht (41)	Poland(Caucasian)	PCR-RFLP	36/64	26	10	0	62	10	51	13	0	115	13	0.102	0.365	6
Chen (47)	China(Asian)	PCR-RFLP	1,022/1,039	781	217	24	1,779	265	793	227	19	1,813	265	0.128	0.557	7
−238G/A				GG	GA	AA	G	A	GG	GA	AA	G	A			
Seung Jo (43)	Korea(Asian)	TaqMan	38/55	36	2	0	74	2	51	4	0	106	4	0.036	0.779	6
Elhawary (48)	Egypt(African)	PCR-RFLP	120/100	82	20	18	184	56	70	30	0	170	30	0.150	0.077	6
Chen (47)	China(Asian)	PCR-RFLP	1,022/1,039	802	202	18	1,806	238	913	119	7	1,945	133	0.064	0.155	7
−857C/T				CC	CT	TT	C	T	CC	CT	TT	C	T			
Mailaparambil (42)	Germany(Caucasian)	PCR-RFLP	44/108	33	6	5	72	16	93	15	0	201	15	0.069	0.438	6
Seung Jo (43)	Korea(Asian)	TaqMan	38/55	22	16	0	60	16	38	15	2	91	19	0.173	0.734	6
Kun (49)	China(Asian)	PCR	31/15	29	0	2	58	4	5	3	7	13	17	0.567	0.021	5
Yan (50)	China(Asian)	PCR	36/14	21	8	7	50	22	7	4	3	18	10	0.357	0.157	6
1,031 T/C				TT	TC	CC	T	C	TT	TC	CC	T	C			
Mailaparambil (42)	Germany(Caucasian)	PCR-RFLP	44/107	25	19	0	69	19	69	36	2	174	40	0.187	0.268	6
Seung Jo (43)	Korea(Asian)	TaqMan	38/55	28	9	1	65	11	38	16	1	92	18	0.164	0.641	6

NOS, Newcastle-Ottawa Scale; MAFs, minor allele frequencies; HWE, Hardy-Weinberg Equilibrium; NA, not available; PCR, polymerase chain reaction; RFLP, restriction fragment length polymorphism.

### Quality of studies

Evaluating the quality of studies included in a meta-analysis requires a thorough assessment of various factors, such as study design, sample size, ethnic diversity, genotyping methodologies, HWE, MAFs, and potential biases. The appraisal utilizing the NOS revealed variability in quality scores, indicating differences in methodology. Studies by Lin (2005) and Chen (47) stood out with the highest ratings of 7 stars, reflecting robust methodologies, while others, such as Mailaparambil (42), Seung Jo (43), Szpecht (41), and Elhawary (48), received 6 stars, suggesting solid approaches with some limitations. Conversely, Kun (49) and Yan (50) scored lower, highlighting areas needing improvement. Larger sample sizes, like that of Chen (47) with 1,022 cases, enhance statistical reliability, while smaller studies, such as Kun (49) with just 31 cases, may yield less dependable results. The inclusion of diverse ethnic populations can improve generalizability, although an overrepresentation of certain groups can limit applicability to others. Reliable genotyping methods, adherence to HWE, and expected MAFs contribute to data quality, with significant discrepancies in case-control ratios potentially introducing bias. Transparency in reporting participant characteristics and conflicts of interest is essential for evaluating study quality. Ultimately, while Chen (47) demonstrates strengths in methodology, the weaknesses in studies like Kun (49) and Elhawary (48) must be carefully weighed against the overall findings in the meta-analysis.

### HWE

To evaluate the HWE across multiple studies, observed genotype frequencies were compared to expected frequencies under HWE. A *p*-value below 0.05 typically indicates significant deviation from equilibrium. The studies by Lin (2005) in Taiwan, Mailaparambil (42) in Germany, Seung Jo (43) in Korea, Szpecht (41) in Poland, and Chen (47) in China for −308G/A reported *p*-values well above 0.05, suggesting they likely maintain HWE. In contrast, Elhawary (48) in Egypt for −238G/A had a *p*-value of 0.077, indicating possible deviation, while Seung Jo (43) and Chen (47) for the same polymorphism remained consistent with HWE. For −857C/T, only the Kun (49) study in China reported a significant deviation (*p*-value = 0.021), while the other studies remained non-significant. Lastly, for the 1,031 T/C polymorphism, both Mailaparambil (42) and Seung Jo (43) showed non-significant *p*-values, indicating consistency with HWE. Overall, most studies align with HWE, with the exception of the notable deviation observed in Kun (49).

### Quantitative synthesis

**Figure 2:**
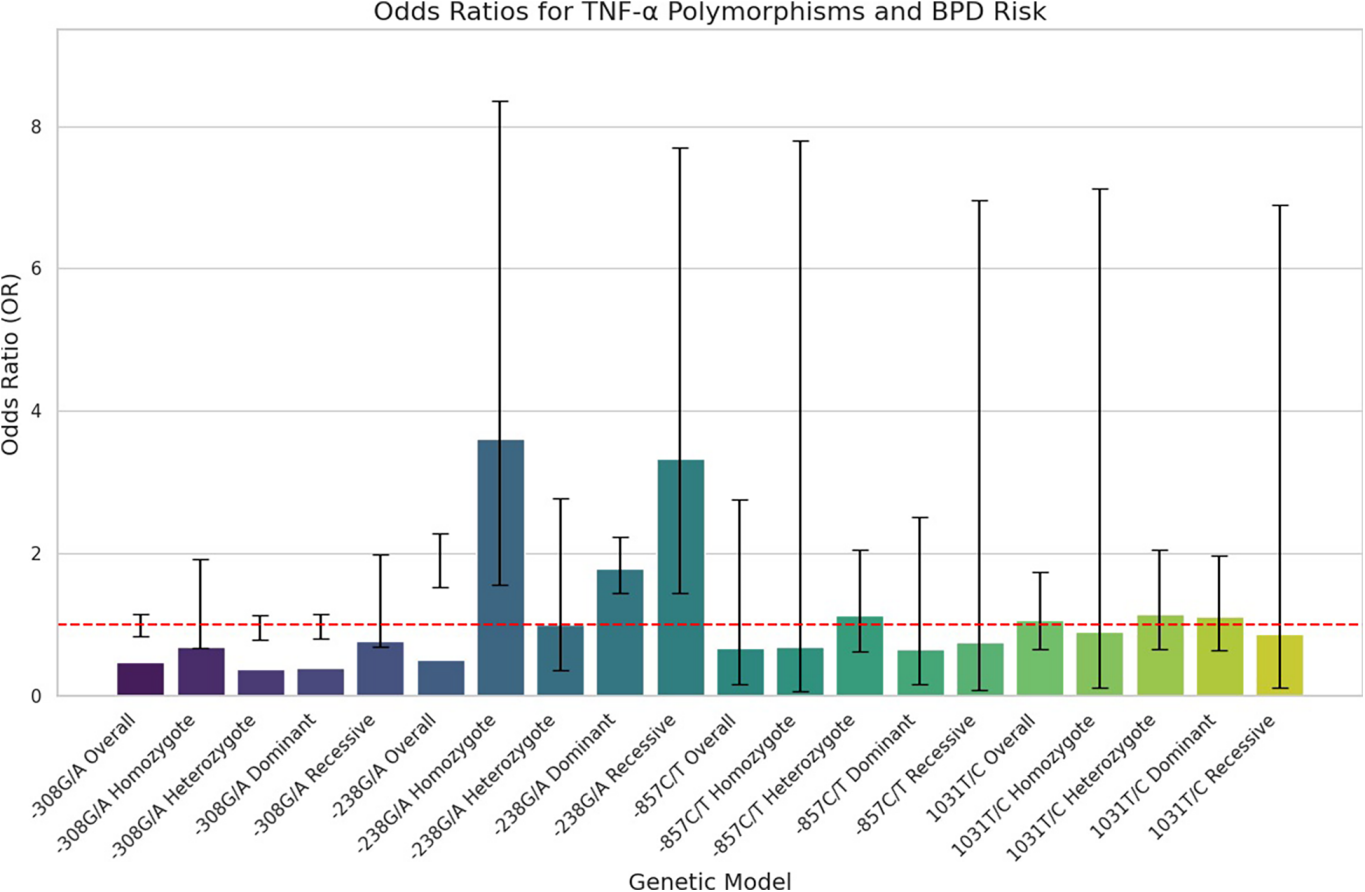
Forest plots illustrating the association between TNF-α polymorphism and BPD risk in preterm infants. Horizontal lines represent 95% CIs for the OR of specific genetic models, with blue circles indicating point estimates of the OR, and a dashed vertical line at OR = 1 for reference.

The key findings on the correlation between TNF-α polymorphisms and BPD susceptibility are presented in Table 2. The analysis of TNF-α polymorphisms in relation to BPD susceptibility revealed significant findings, particularly for the −238G/A polymorphism, which demonstrated strong associations across various genetic models. Specifically, the allele comparison (A vs. G) showed an OR of 1.866 (95% CI 1.527–2.281, *p* ≤ 0.001), while the heterozygote model (AA vs. GG) indicated an OR of 3.609 (95% CI 1.560–8.349, *p* = 0.003). Additionally, both the dominant and recessive models presented robust associations with ORs of 1.791 (95% CI 1.439–2.229, *p* ≤ 0.001) and 3.329 (95% CI 1.440–7.695, *p* = 0.005), respectively. In contrast, the −308G/A polymorphism revealed no significant association with BPD, consistently yielding ORs below 1, indicating a potential but statistically insignificant lower risk. The −857C/T polymorphism also lacked significant associations, with low risk suggested by various ORs, and the −1,031 T/C polymorphism similarly showed no significant findings, with all comparisons yielding low ORs. Subgroup analysis highlighted ethnic differences, revealing that the −238G/A polymorphism was associated with a significantly increased risk of BPD in the Asian population (A vs. G: OR 1.896, *p* ≤ 0.001), while Caucasian populations showed no significant associations with the studied polymorphisms. The −857C/T polymorphism exhibited mixed results, suggesting a potential protective effect in some models. These findings underscore the necessity of considering ethnic variations in genetic studies related to BPD susceptibility. Figure 2, plot illustrates the OR for various TNF-α polymorphisms associated with the risk of BPD.

**Table 2:** Summary of pooled risk estimates for the association between TNF-α polymorphisms and BPD risk.

Subgroup	Genetic model	Type of model	Heterogeneity		Odds ratio (OR)				Publication bias	
			I^2^ (%)	P_H_	OR	95% CI	Z_OR_	P_OR_	P_Beggs_	P_Eggers_
−308G/A										
Overall	A vs. G	Fixed	0.00	0.481	0.981	0.836–1.152	−0.232	0.817	1.000	0.907
	AA vs. GG	Fixed	0.00	0.687	1.130	0.668–1.912	0.454	0.649	0.296	0.121
	AG vs. GG	Fixed	6.13	0.372	0.940	0.780–1.133	−0.648	0.517	0.462	0.997
	AA + AG vs. GG	Fixed	3.11	0.389	0.958	0.800–1.147	−0.467	0.641	0.806	0.948
	AA vs. AG + GG	Fixed	0.00	0.771	1.175	0.695–1.983	0.602	0.547	0.296	0.010
Asian	A vs. G	Fixed	16.05	0.304	0.980	0.829–1.160	−0.230	0.818	1.000	0.839
	AA vs. GG	Fixed	0.00	0.547	1.176	0.685–2.022	0.588	0.556	NA	NA
	AG vs. GG	Fixed	36.99	0.205	0.929	0.764–1.130	−0.738	0.461	1.000	0.826
	AA + AG vs. GG	Fixed	33.22	0.224	0.952	0.789–1.149	−0.513	0.608	1.000	0.829
	AA vs. AG + GG	Fixed	0.00	0.703	1.222	0.713–2.095	0.729	0.466	NA	NA
Caucasian	A vs. G	Fixed	8.79	0.295	0.989	0.570–1.715	−0.041	0.968	NA	NA
	AA vs. GG	Fixed	0.00	1.000	0.568	0.061–5.280	−0.497	0.619	NA	NA
	AG vs. GG	Fixed	0.00	0.335	1.062	0.568–1.983	0.187	0.851	NA	NA
	AA + AG vs. GG	Fixed	7.49	0.295	1.026	0.557–1.890	0.082	0.935	NA	NA
	AA vs. AG + GG	Fixed	0.00	1.000	0.599	0.065–5.514	−0.453	0.651	NA	NA
−238G/A										
Overall	A vs. G	Fixed	0.00	0.505	1.866	1.527–2.281	6.098	≤0.001	0.296	0.116
	AA vs. GG	Fixed	59.71	0.115	3.609	1.560–8.349	3.000	0.003	NA	NA
	AG vs. GG	Random	84.37	0.002	1.008	0.367–2.766	0.016	0.987	NA	NA
	AA + AG vs. GG	Fixed	58.05	0.092	1.791	1.439–2.229	5.224	≤0.001	1.000	0.313
	AA vs. AG + GG	Fixed	66.85	0.082	3.329	1.440–7.695	2.814	0.005	NA	NA
Asian	A vs. G	Fixed	19.80	0.264	1.896	1.522–2.363	5.706	≤0.001	NA	NA
	AA vs. GG	Fixed	0.00	1.000	2.927	1.216–7.045	2.397	0.017	NA	NA
	AG vs. GG	Fixed	19.27	0.266	1.895	1.486–2.417	5.154	≤0.001	NA	NA
	AA + AG vs. GG	Fixed	23.71	0.252	1.951	1.540–2.471	5.538	≤0.001	NA	NA
	AA vs. AG + GG	Fixed	0.00	1.000	2.643	1.099–6.355	2.171	0.030	NA	NA
−857C/T										
Overall	T vs. C	Random	90.05	≤0.001	0.679	0.167–2.755	−0.542	0.588	0.308	0.059
	TT vs. CC	Random	78.69	0.003	0.689	0.061–7.788	−0.301	0.763	1.000	0.568
	TC vs. CC	Fixed	58.88	0.063	1.129	0.622–2.051	0.399	0.690	0.089	0.010
	TT + TC vs. CC	Random	82.98	0.001	0.653	0.169–2.518	−0.619	0.536	0.308	0.028
	TT vs. TC + CC	Random	76.01	0.006	0.753	0.081–6.964	−0.250	0.802	1.000	0.608
Asian	T vs. C	Random	89.47	≤0.001	0.402	0.074–2.175	−1.058	0.290	0.296	0.171
	TT vs. CC	Fixed	59.83	0.083	0.243	0.081–0.763	−2.431	0.015	1.000	0.917
	TC vs. CC	Random	72.59	0.026	0.555	0.095–3.239	−0.654	0.513	0.296	0.030
	TT + TC vs. CC	Random	86.17	0.001	0.394	0.056–2.755	−0.938	0.348	0.296	0.162
	TT vs. TC + CC		52.05	0.124	0.309	0.105–0.907	−2.137	0.033	1.000	0.833
1,031 T/C										
Overall	C vs. T	Fixed	0.00	0.531	1.065	0.652–1.738	0.251	0.802	NA	NA
	CC vs. TT	Fixed	0.00	0.668	0.895	0.112–7.124	−0.105	0.916	NA	NA
	CT vs. TT	Fixed	11.24	0.288	1.151	0.648–2.044	0.481	0.630	NA	NA
	CC + CT vs. TT	Fixed	0.00	0.358	1.123	0.638–1.976	0.402	0.688	NA	NA
	CC vs. CT + TT	Fixed	0.00	0.595	0.873	0.111–6.892	−0.129	0.898	NA	NA

### Heterogeneity test

The evaluation of heterogeneity in meta-analyses revealed differing levels across various polymorphisms. While the −308G/A variant showed low to no heterogeneity, indicating strong consistency among studies, the −238G/A polymorphism displayed moderate to high heterogeneity, especially in the AA vs. GG comparison (I^2^ = 59.71%) and AG vs. GG (I^2^ = 84.37%), suggesting significant variability that warrants further investigation. Similarly, the −857C/T polymorphism exhibited high heterogeneity overall (I^2^ = 90.05%) and across specific comparisons such as TT vs. CC (I^2^ = 78.69%), implying notable differences among studies that require careful interpretation. Conversely, the 1,031 T/C polymorphism demonstrated no heterogeneity in any comparisons, indicating consistent outcomes across studies. These findings underline the high heterogeneity of the −238G/A and −857C/T polymorphisms, which may be influenced by factors like participant characteristics and study designs, while the low heterogeneity observed in the −308G/A and 1,031 T/C polymorphisms suggests more reliable results. The discrepancies in the meta-analyses of the −238G/A and −857C/T polymorphisms can be linked to variations in study populations, methodological designs, and outcome measures, including demographic factors such as age, sex, and ethnicity. These differences may result in diverse genetic interactions and influences on health outcomes. Moreover, variations in study designs, sample sizes, recruitment strategies, data collection methods, and the types of outcomes assessed can further contribute to inconsistencies. This variability emphasizes the need for standardized protocols and a deeper exploration of participant characteristics to improve the robustness and generalizability of findings in genetic association studies.

### Sensitivity analysis

**Figure 3:**
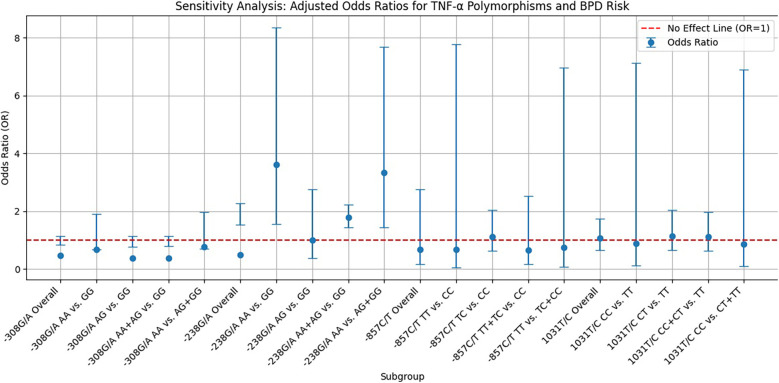
Funnel plot displaying sensitivity analysis results on the relationship between TNF-α polymorphisms and BPD risk. Blue dots represent the OR for specific genetic models, vertical error bars indicate 95% CIs, and a red dashed line at OR = 1 shows no effect.

As shown in Figure 3, the sensitivity analysis of TNF-α polymorphisms in relation to BPD risk reveals significant genetic influences that complicate our understanding of susceptibility to this condition. Most genetic models indicate ORs below 1, suggesting a potential protective effect; however, the −238G/A homozygote model demonstrates a notable OR of 3.609, signaling an increased risk for developing BPD linked to this genotype. This finding highlights a specific genetic variant that may serve as a biomarker for higher risk, potentially aiding clinical monitoring and intervention. In contrast, the −857C/T allele model indicates a protective effect with an ORsof 0.679, although its wide confidence interval includes values above 1, introducing uncertainty about its clinical relevance. This underscores the need for further research to confirm the associations and clarify the implications of these genetic polymorphisms. The variability in CIs across different models accentuates the complexity of genetic contributions to BPD risk, advocating for larger, more meticulously designed studies to explore these relationships more comprehensively. Until definitive evidence is established, the clinical significance of the protective effects remains ambiguous, emphasizing the necessity of ongoing research into the genetic underpinnings of BPD risk to enhance prevention and treatment strategies.

### Publication bias

**Figure 4:**
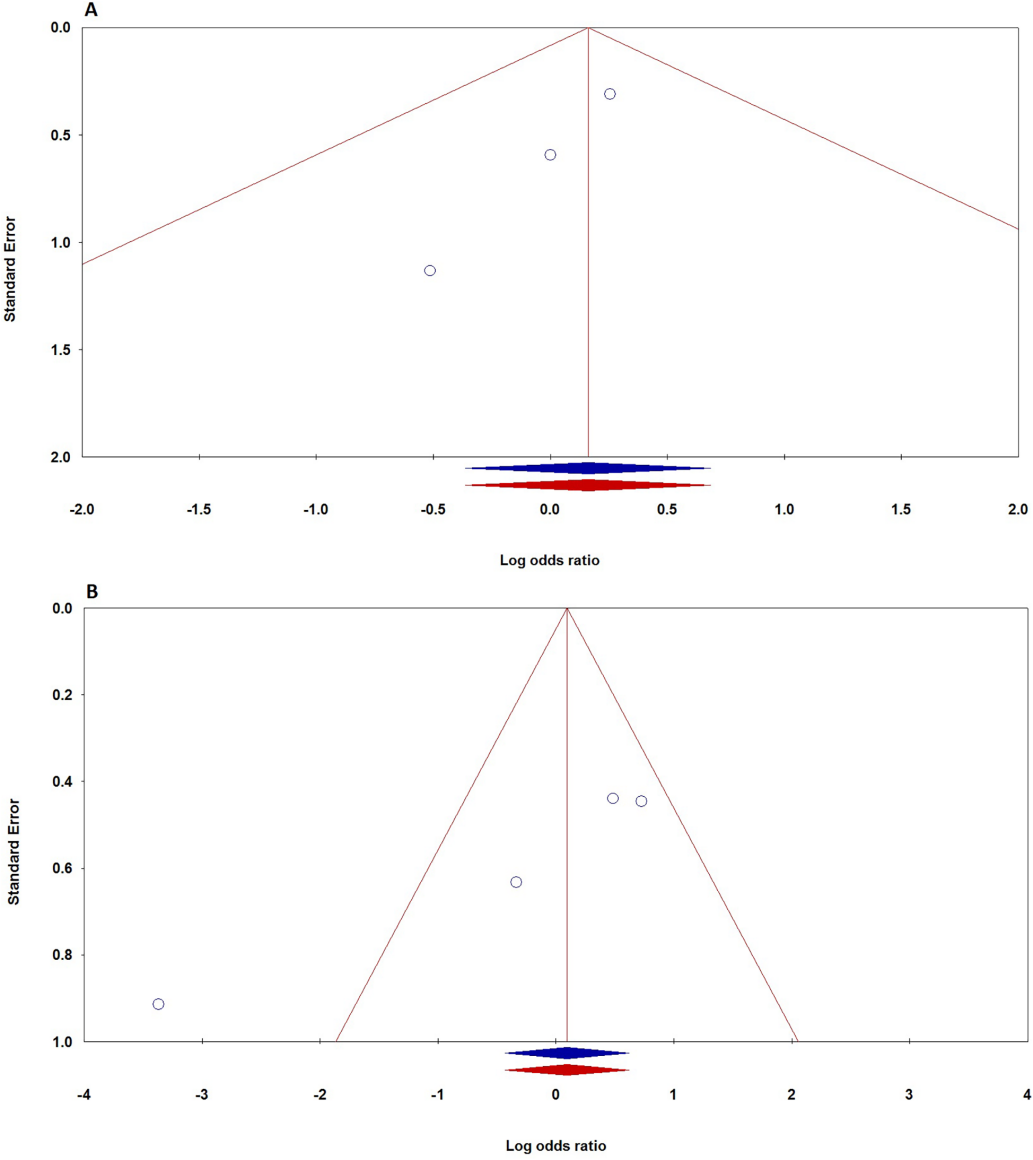
Funnel plot demonstrating publication bias in studies on TNF-α polymorphisms and BPD risk in preterm infants: **A**: −308G/A (recessive model: AA vs. AG + GG); **B**: −857C/T (dominant model: TT + TC vs. CC). Plots are shown before (blue) and after (red) the “Trim-and-Fill” method application.

**Figure 5:**
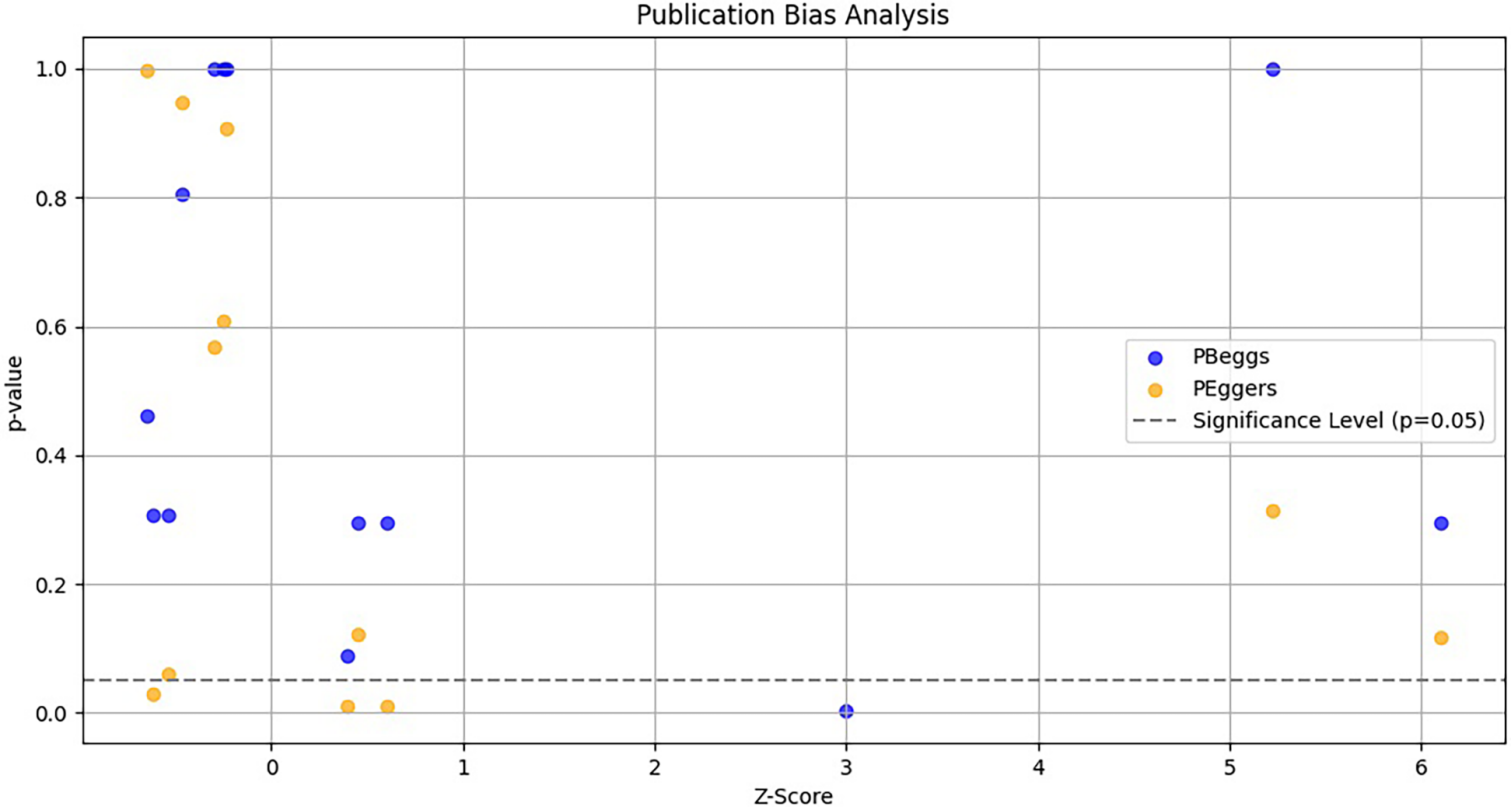
Funnel plot depicting the relationship between Z-scores and *p*-values for publication bias tests (Begg's pBeggs and egger's pEggers) related to TNF-α polymorphisms and BPD risk. The *x*-axis shows Z-scores indicating effect strength, the *y*-axis displays corresponding *p*-values, and a dashed horizontal line at *p* = 0.05 indicates the threshold for statistical significance.

To evaluate potential publication bias in studies on TNF-α polymorphisms and BPD, we utilized Egger's test and Begg's funnel plots. Our analysis revealed no significant publication bias for TNF-α polymorphisms across various genetic models, as demonstrated by high *p*-values from both the PBeggs and PEggers tests for the −308G/A polymorphism, indicating minimal bias. However, while the −238G/A polymorphism showed no bias in the A vs. G model, the AA vs. GG model exhibited a significant PEggers value, suggesting the need for caution. For the −857C/T polymorphism, the TC vs. CC model showed a concerning PEggers value, though other models generally reflected favorable PBeggs results. Overall, our findings suggest that most association estimates are reliable and unbiased, with specific caution advised for the −238G/A and −857C/T polymorphisms due to potential publication bias. Notably, the AA vs. AG + GG model of the −308G/A polymorphism (Figure 4A) and the TC vs. CC model of the −857C/T polymorphism (Figure 4A) both showed significant P_Eggers_ values of 0.010. The relationship between Z-scores and *p*-values from publication bias tests indicates that most PBeggs points cluster above the significance line (Figure 5), showing limited bias; however, several PEggers points approach the significance threshold, warranting further scrutiny of these borderline cases. The implications of publication bias could lead to an inflated perception of the association between TNF-α polymorphisms and BPD risk, particularly as studies with positive results are more likely to be published. While most association estimates appear reliable, the significant PEggers values indicate potential underreporting of studies with non-significant results, which could mislead researchers and clinicians about genetic risk factors. Thus, despite the overall confidence in association estimates, these identified biases underscore the necessity for further investigation into borderline cases to better understand the true effects of these polymorphisms on BPD risk and their clinical significance.

### MAF

The mean MAF for various polymorphisms was calculated from data across multiple studies involving different countries and ethnicities. The overall mean MAFs were −308G/A at 0.1584, −238G/A at 0.0833, −857C/T at 0.2665, and 1,031 T/C at 0.1755. By country, Taiwan had the highest mean for −308G/A at 0.214; Germany averaged 0.1413 across three polymorphisms, Korea had 0.1138, Poland recorded 0.102, China reached 0.218, and Egypt stood at 0.150. Ethnic breakdowns revealed an overall mean of 0.2043 for Asians, 0.1303 for Caucasians, and 0.150 for Africans. This analysis underscores significant MAF differences across populations, highlighting the need to consider geographic and ethnic contexts in genetic research.

## Discussion

The human TNF-α gene is located in 6 p21.3, consists of 3 introns and 4 exons, and spans 2.76-kb DNA (51). It has a variety of biological activities produced by monocytes and macrophages are the main mediators involved in inflammation and tissue cell damage and are closely related to the occurrence of respiratory diseases (52–54). Emerging evidence shows that TNF-α along with other cytokines is an important mediator in the pathogenesis of BPD. Comparative data on cytokine profiles in serum and tracheobronchial aspirate fluid concentrations in infants with BPD revealed elevated levels of TNF-α, IL-8, IL-1β, IL-6, monocyte chemo-attractant proteins (MCPs) and macrophage inflammatory proteins (MIP), and decreased level of IL-10, which indicating dysregulation of pro- and anti-inflammatory factors in BPD (3, 55, 56). Ehrhardt et al., studied whether the lack of TNF-α could enhance the inflammatory response in neonatal lungs during mechanical ventilation, they found that TNF-α was related to BPD. Moreover, their results showed a critical balance between TNF-α and TGF-β signaling in the developing lung, and underscore the considerable importance of these central pathways in the development of BPD in premature neonates (37, 57, 58). These data indicate that the evaluation of cytokines concentrations has great promise as biomarkers for the prediction and early treatment of BPD in preterm neonates. It seems that TNF-α might be involved in the pathogenesis of BPD in neonates via two different pathways. First, it triggers a series of various inflammatory responses in infants with BPD. Secondly, it can inhibit the generation of fibrosis by suppressing the TGF-β1 induced connective tissue growth factor (CTGF) synthesis by fibroblastic cells. Animal model studies showed that over-expression of TNF-α in the lungs causes an interference with alveolarization. On the other hand, genetic epidemiological examinations revealed that genetic variations at the TNF-α gene, which produces lower levels of TNF-α, are correlated inversely with the severity of BPD. Despite these findings plasma levels of TNF-α and its genetic variants have not been consistently correlated with BPD and its clinical outcomes (11).

Over the past decade, numerous studies have investigated the relationship between TNF-α polymorphisms and BPD risk in preterm infants across diverse populations, including those from China, Taiwan (46), Korea (43), Poland (41), Egypt (48), and Germany (42). The findings have been inconsistent regarding TNF-α's association with BPD incidence, particularly among different ethnic groups, highlighting the need for further research. Our pooled data indicate a significant link between the TNF-α −238G/A polymorphism and overall BPD risk, whereas no significant correlations were established for the −308G/A, −857C/T, and 1,031 T/C polymorphisms. Chauhan et al. conducted a meta-analysis of six cohorts comprising 804 preterm infants, revealing no significant association between the TNF-α −308G/A polymorphism and BPD risk in extremely premature infants, though they did not evaluate control subjects, which raises questions about their conclusions due to a smaller number of included studies (39). Elhawary et al. studied the TNF-α −238G/A polymorphism in 220 premature infants and reported a significant correlation with BPD risk among Egyptian infants, noting that the mutant allele was prevalent in those with moderate and severe BPD compared to mild cases. Chen et al. examined 14 polymorphisms across multiple cytokines in 1,022 BPD cases and 1,039 controls, finding that only the TNF-α −238G/A polymorphism demonstrated a significant association with BPD risk in Han Chinese newborns (47). Strassberg et al. assessed five TNF-α polymorphisms in 105 neonates, discovering no correlation with BPD risk (59), while Kazzi et al. suggested a potential protective role of the TNF-α −238 polymorphism in low birth weight premature neonates (38). Meiqi et al. reported no significant mutations in the TNF-α gene among Han and Mongolian preterm infants, indicating varying influences on the impact of TNF-α polymorphisms and acknowledging limitations in their study's design (60). Suprun et al. explored the roles of MMP-12, TNF-α, and IL-6 genes in children with chronic bronchopulmonary diseases, suggesting that certain polymorphisms might contribute to the pathophysiology of BPD (61), while Yu et al. found significant associations of other polymorphisms, specifically TNF-α −857C/T and TLR-10 rs11096955, with BPD risk among Han premature infants (62).

The literature review offers a thorough examination of various studies investigating the link between TNF-α polymorphisms and the risk of BPD in preterm infants, highlighting key findings and methodological considerations. It underscores the diversity of studies conducted across different populations, which enhances the discussion surrounding the potential role of TNF-α polymorphisms but also raises concerns about the generalizability of these results. The review points to contradictory findings, such as the conflicting conclusions drawn by Elhawary et al. (48) and Chauhan et al. (39), which challenge the stability of SNP associations amidst variations in sample size and methodology. Limitations related to sample sizes, particularly in studies lacking adequate control groups, may impede the identification of true associations. The notion that specific genotypes, particularly TNF-α −857C/T and −238 polymorphisms, may confer protection against BPD opens avenues for future research, although careful consideration of confounding variables is crucial. Investigating these genetic factors could deepen our understanding of BPD and inform targeted therapies for at-risk populations. Furthermore, exploring the interaction between genetic and environmental influences will be vital for effective BPD prevention strategies. The review also emphasizes the need for methodological rigor, particularly in matching cases and controls, to enhance the reliability of findings. Acknowledging environmental influences on TNF-α expression is essential, especially in heterogeneous populations. A recurring theme is the call for larger, more diverse cohorts to solidify the associations between TNF-α polymorphisms and BPD risk, with suggestions for conducting meta-analyses to provide clearer insights. Finally, the broader implications of this research highlight the potential connections between TNF-α polymorphisms and chronic lung diseases, reinforcing the need for continuous exploration of genetic factors that affect the respiratory health of premature infants.

To the best of the authors’ knowledge, this is the first meta-analytic study investigating the correlation between TNF-α polymorphisms and the development of BPD, utilizing a comprehensive database search. However, several limitations should be noted regarding this meta-analysis. Firstly, a primary limitation is the scarcity of available case-control studies, with only two studies focusing on the 1,031 T/C polymorphism. This limited dataset makes it difficult to draw confident conclusions regarding the association between these polymorphisms and BPD risk. Secondly, there is a notable lack of clear evidence regarding the association of TNF-α polymorphisms with BPD in African populations, leaving this aspect of the gene's effect on BPD susceptibility unclear. Thirdly, mild publication bias may have influenced the findings, as only a small number of relevant studies were identified from diverse populations. Therefore, the results related to TNF-α polymorphisms should be interpreted with caution. Additionally, all selected studies were published in either English or Chinese, introducing a potential language bias that may have resulted in missed publications. Lastly, the analysis relied on crude estimations of TNF-α polymorphism associations with BPD, without accounting for the impact of confounding variables such as gender, gestational age, birth weight, severity of BPD, mortality rates, intrauterine growth restriction (IUGR), Apgar scores, mode of delivery, intrauterine infections, and the necessity for ventilatory support. Moreover, potential interactions, such as gene-gene and gene-environment relationships, were not thoroughly examined. Consequently, the conclusions drawn from this study necessitate validation through larger-scale studies encompassing diverse populations.

In a nutshell, our pooled analysis found a significant association between the TNF-α −238G/A polymorphism and BPD susceptibility, while the −308G/A, −857C/T, and −1,031 T/C polymorphisms showed no significant links. However, this meta-analysis has limitations, including potential publication bias and heterogeneity due to the small number of studies, which may affect the reliability of our conclusions. Furthermore, population variability adds complexity to interpreting the relationship between TNF-α polymorphisms and BPD risk. Future research with larger, diverse ethnic samples is essential to explore gene-gene and gene-environment interactions and provide deeper insights into the mechanisms of BPD development.

## Data Availability

The original contributions presented in the study are included in the article/Supplementary Material, further inquiries can be directed to the corresponding author.
